# A modifier screen in the *Drosophila *eye reveals that *aPKC *interacts with *Glued *during central synapse formation

**DOI:** 10.1186/1471-2156-10-77

**Published:** 2009-11-30

**Authors:** Lisha Ma, Louise A Johns, Marcus J Allen

**Affiliations:** 1 Cell and Developmental Biology Group, School of Biosciences, University of Kent, Canterbury. UK

## Abstract

**Background:**

The *Glued *gene of *Drosophila melanogaster *encodes the homologue of the vertebrate p150^Glued ^subunit of dynactin. The *Glued*^1 ^mutation compromises the dynein-dynactin retrograde motor complex and causes disruptions to the adult eye and the CNS, including sensory neurons and the formation of the giant fiber system neural circuit.

**Results:**

We performed a 2-stage genetic screen to identify mutations that modified phenotypes caused by over-expression of a dominant-negative Glued protein. We screened over 34,000 flies and isolated 41 mutations that enhanced or suppressed an eye phenotype. Of these, 12 were assayed for interactions in the giant fiber system by which they altered a giant fiber morphological phenotype and/or altered synaptic function between the giant fiber and the tergotrochanteral muscle motorneuron. Six showed interactions including a new allele of *atypical protein kinase C *(*aPKC*). We show that this cell polarity regulator interacts with *Glued *during central synapse formation. We have mapped the five other interacting mutations to discrete chromosomal regions.

**Conclusion:**

Our results show that an efficient way to screen for genes involved in central synapse formation is to use a two-step strategy in which a screen for altered eye morphology precedes the analysis of central synaptogenesis. This has highlighted a role for *aPKC *in the formation of an identified central synapse.

## Background

During the development of a neural connection the axon of the growing neuron has to make morphogenic changes to form the presynaptic apparatus needed for efficient synaptic function once it has reached its target cell. This process involves reception of signals by the presynaptic cell followed by precise rearrangements of the cytoskeleton to direct changes in cell shape and control the formation of the presynaptic apparatus (see [1,2] for reviews).

The giant fiber system (GFS) is a unique neural circuit that contains several of the few identified central synapses in *Drosophila *and includes the largest in the fly between the giant fiber (GF) interneuron and the leg extensor muscle motorneuron, the tergotrochanteral motorneuron (TTMn), the GF-TTMn synapse [3]. Several studies using over-expression of dominant-negative transgenes, or homozygous adult viable mutations, have recently shed light on signaling mechanisms during the formation of the GF-TTMn synapse. These include the receptors Semaphorin 1a and Roundabout [4,5]; the L-1 type cell-adhesion molecule Neuroglian [6]; the endocytotic and ubiquitin machinery [7-10]; the small GTPase DRac1 [11], and the transcription factor Ken [12]. However, the precise mechanisms by which these integrate during synaptogenesis are yet to emerge.

*Glued *encodes the largest subunit of the retrograde motor dynein-activating complex dynactin [13,14]. The *Glued*^1^(*Gl*^1^) mutation results in a truncated protein product [15] that disrupts the dynein-dynactin complex by binding to dynein and microtubules but fails to bind to cargoes [16]. Mutants have both visual and CNS defects [17-19]. Glued has a key role in formation of the GF presynaptic bend which may involve local cytoskeletal dynamics and rearrangements [20]. Forward genetic screens to identify gene products involved in post-mitotic neural differentiation can be problematic as many of these genes are plieotropic and will have vital functions earlier in development, thus preventing mutants from being recovered. Moreover, many of the genes may well have several functions during differentiation of a single neuronal type. Consequently, mutants, therefore, will exhibit phenotypes that are difficult to interpret. Indeed, this has been shown for the dynein-dynactin complex in the formation of mushroom body neurons [21]. The eye is not required for either viability or fertility of the adult and genetic disruptions targeted to the eye have been exceptionally useful in deducing signaling pathways, for example the *sevenless *pathway [22], and also in identifying mutant alleles that would cause lethality earlier in development if they were to be expressed throughout the organism [23-25]. This coupled with the fact that it has been estimated that two thirds of the vital genes within the *Drosophila melanogaster *genome (~2,500) are involved in its development [26], makes the eye invaluable in a primary screen for genes with additional roles in processes such as neural differentiation.

In this study we have undertaken a genetic modifier screen in the adult eye and isolated 12 mutations that either dominantly enhance or suppress a phenotype caused by over-expression of a dominant-negative form of the Glued protein (Gl^DN^). We assayed these mutations for additional interactions with *Glued *in the GFS and found that 6 show interactions. Mapping of each modifier mutation is presented. One of the suppressors is an allele of *aPKC*, and we found that other aPKC mutant alleles exhibit suppression of the synaptic phenotype caused by over-expression of Gl^DN^.

## Results

### Screening for modifiers of a truncated Glued (Gl^DN^) over-expression eye phenotype

*Gl*^1 ^is a true dominant-negative mutation and the effects of the truncated product produced are dose-sensitive [17,20,27]. We previously exploited this by generating a UAS-Gl^DN ^transgene to over-express this "poison subunit" using the GAL4-UAS system [28] and showed that disruption of retrograde motor function resulted in synaptic defects in the GFS [20]. Our aim was to identify genes that acted with *Glued *during GFS formation in the developing CNS. Direct screening for alterations of an adult neural phenotype is problematic and extremely labor-intensive because it would involve an F2 screen. Individual mutant stocks would need to be made and crossed into the appropriate mutant background followed by dissection and staining of the adult CNS.

**Figure 1:**
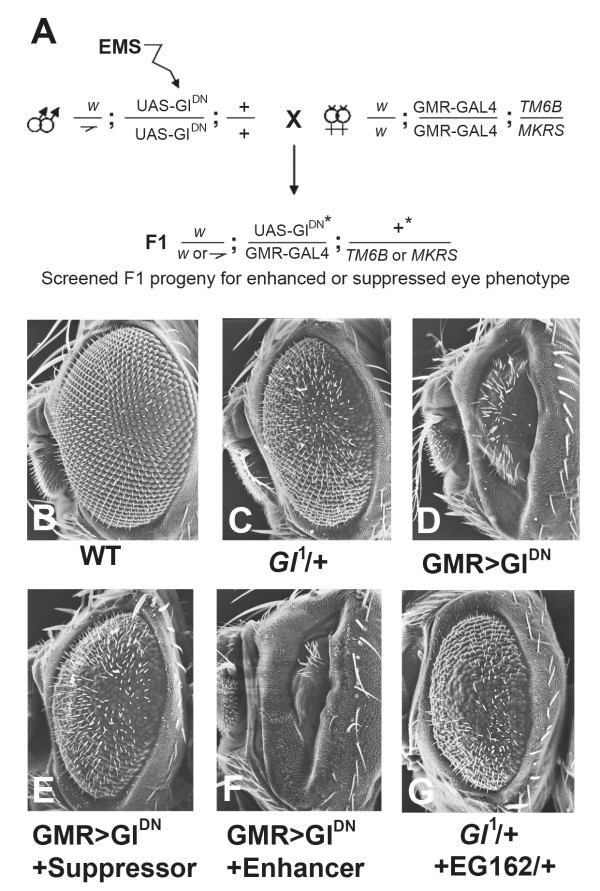
**The Gl^DN ^sensitized screen in the adult eye**. (A) Schematic of the screen in which UAS-Gl^DN ^males were mutagenized and crossed to GMR-GAL4 virgin females. In the F1 generation the flies were scored for any enhancement or suppression of the eye phenotype caused by over-expression of Gl^DN^. (B-D) Scanning electron micrographs of adult eyes. (B) w; UAS-Gl^DN^, showing a wild type eye with a regular array of ommatidia and bristles. (C) *Gl*^1^/+ exhibiting a roughened, smaller eye. (D) w; GMR-GAL4/UAS-Gl^DN ^(abbreviated to GMR>Gl^DN ^throughout) showing the much reduced and disorganized eye phenotype used as the basis for the screen. (E) shows the effects of a dominant suppressor (SG13/+) on the phenotype in (D), and (F) shows the effects of a dominant enhancer (EG37/+). (G) *Gl*^1^/+; EG162/+, showing an enhanced phenotype than for *Gl*^1^/+ alone depicted in (C).

We therefore reasoned that since *Gl*^1 ^affects the development of the eye, monitoring the eye phenotype in a primary F1 screen would provide an excellent read-out for defining interacting loci. Using the eye-specific GMR-GAL4 line to target truncated Glued (Gl^DN^) resulted in the generation of adult flies possessing smaller eyes with fused ommatidia and miss-arrayed eye bristles. As expected, this was a more severe phenotype than that seen in *Gl*^1 ^mutants (Figure 1C &1D). This disruption provided a sensitized background in which to base an F1 screen on adult eyes to isolate novel mutations that altered this phenotype. We performed an EMS screen for second-site modifiers that dominantly enhanced or suppressed the over-expression phenotype in the eye (Figure 1A; see materials and methods). We screened over 34,000 flies and obtained both suppressors (Figure 1E) and enhancers (Figure 1F) that reproducibly altered the eye phenotype. We recovered nine lines with homozygous lethal mutations on the second chromosome and seven lines with homozygous lethal mutations on the third chromosome that were either enhancers of *Glued *(EGs) or suppressors of *Glued *(SGs). The lethal mutations were most likely the dominant modifiers of the eye phenotype but we could not rule out the posibility of second-site mutations on the chromosomes that were responsible for altering the phenotype. The lines were crossed *inter se *for complementation. All mutations showed complementation illustrating each of the 16 mutations map to independent loci (data not shown). Twelve of these were analyzed further (see below).

### Identification of mutations that interact with *Gl*^1 ^in the adult eye

**Figure 2:**
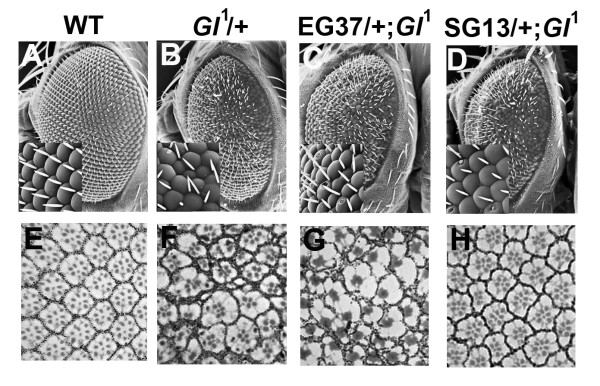
**An enhancer and a suppressor interact with *Gl*^1 ^in the adult eye**. (A-D) Scanning electron micrographs of adult eyes. (A) Wild type. (B) *Gl*^1^/+, (C) EG37/+;*Gl*^1^/+, both exhibiting a roughened, smaller eye. (D) SG13/+;*Gl*^1^/+ showing a slight amelioration of the *Gl*^1 ^phenotype. Insets are higher magnifications. (E-H) Tangential sections of adult eyes. (E) Wild type showing the regular pattern of ommatidial assembly and the stereotyped trapezoidal pattern of the rhabdomeres of the photoreceptor cells (R1-R7). (F) *Gl*^1^/+ eye showing disordered and irregular shaped ommatidia often with aberrant numbers of rhabdomeres. (G) The disorganization is exacerbated in the presence of EG37/+ with the rhabomeres often fused. (H) In the presence of SG13/+ the ommatidia show a more ordered, regular, pattern with each ommatidia often having the correct array of rhabdomeres (compare with E and G).

Our sensitized screen allowed us to isolate mutations that reproducibly dominantly enhanced or suppressed the eye phenotype caused by over-expression of Gl^DN^. From these lines we wished to identify which mutations dominantly interacted with *Glued*, rather than simply up- or down-regulated the GAL4/UAS system or interacted with the synthetic GMR element which can cause disruptions if homozygous or if the temperature is raised [29]. To do this we tested whether these mutations could dominantly interact with the *Gl*^1 ^allele by making flies heterozygous for both *Gl*^1 ^and the novel EG or SG mutations and identifying subsequent alterations of the *Gl*^1 ^eye phenotype. The *Gl*^1 ^eye phenotype was clearly exacerbated by the enhancer EG162 (Figure 1C &1G). Other mutations caused more subtle alterations of the *Gl*^1 ^adult eye phenotype that were either variable or could not be distinguished when viewed under a dissecting microscope. The eyes of *Gl*^1^/+ individuals show irregular ommatidia and bristle orientation as revealed by SEM (compare Figure 2B to 2A). In addition retinal sections reveal a variable number of rhabdomeres in each ommatidium, which are often reduced in size, as well as clear disruption of the accessory cells. As previously reported, these affects produce a distortion in the overall shape of the ommatidia (Figure 2F), [17]. To determine whether we had isolated interacting mutations we examined whole eyes by SEM and retinal sections by light microscopy from the eyes of *Gl*^1^/+ flies and those transheterozygous with an enhancer (EG37) and a suppressor (SG13). When EG37 was introduced into the *Gl*^1 ^background (EG37/+; *Gl*^1^/+) we saw an increase in bristles and an increase in ommatidial fusion and disorganization (Figure 2C). Retinal sections of the eyes from these flies revealed increased disruptions of accessory cells and fused rhabdomeres (Figure 2G). The number of rabdomeres per ommatidium was 4.43 ± 1.11 (*n *= 195, P < 0.001) compared to those from flies containing *Gl*^1^/+ alone which had 5.03+1.28 (*n *= 119). We also sometimes saw holes between some ommatidia in the sections (data not shown). With SG13 in the *Gl*^1 ^background (SG13/+; *Gl*^1^/+), the ommatidia and bristles became more ordered than is seen in *Gl*^1 ^alone (Figure 2, compare D [inset] with B [inset]) and the sections revealed a return to a more hexagonal lattice of accessory cells and trapezoidal pattern of the rhabdomeres (Figure 2H). These contained an average of 5.89 ± 1.13 (n = 195, P < 0.001) rhabdomeres per ommatidium. These data indicated that, for at least two of our mutations, we had isolated loci that genetically interact with *Glued *during eye development. A third suppressor, SG46, was also analysed in this way and also showed supression of the *Gl*^1 ^eye phenotype (data not shown).

### Mutations that interact with *Glued *in the Giant Fiber System and alter axon morphology

The presynaptic terminal of the GF-TTMn synapse is a distinctive bend at the end of the GF axon closely apposed to the TTMn dendrite [30]. Several studies have reported that this bend is often absent or altered when the two neurons fail to form a proper synapse [4,6,7,9,11,20,31]. Since our main aim was to identify genes involved in synapse formation within the GFS we next performed morphological analysis of the giant fiber neurons in adult flies carrying an EG or SG mutation and with disrupted *Glued *function.

**Figure 3:**
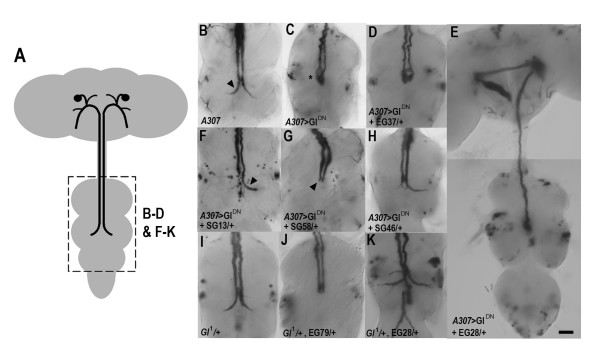
**Mutations that interact with *Glued *and alter GF axon morphology**. (A) Schematic of the adult CNS with the GFs indicated. Hatched box indicates the approximate area of the ventral ganglia depicted in B-D & F-K. (B-K) Dissected adult nervous systems stained for LacZ expression. (B) UAS-LacZ; *A307 *control showing normal GFs with their characteristic bends in the mesothoracic neuromere (arrowhead) where the GF synapses with the TTMn. (C) A fly also expressing UAS-Gl^DN ^exhibits swollen, bendless, axon tips (asterisk). (D) The introduction of EG37/+ did not noticeably enhance the swollen axon phenotype. (E) The whole nervous system is shown for this preparation in which the introduction of EG28/+ results in a more severe phenotype with one GF remaining in the brain. (F-H) Three different suppressors showing amelioration of the swollen axon phenotype. Note that GFs from specimens carrying SG13/+ or SG46/+ show normal diameter axons and sometimes a terminal bend (arrowhead) and that the GFs from specimens carrying SG58/+ also exhibit normal diameter axons. (I) A fly carrying the *Gl*^1 ^mutation exhibits wild type GF morphology. (J&K) preparations from flies carrying *Gl*^1 ^and an enhancer sometimes showed enhancement or altered GF phenotypes (see Table 1 and text for details). Scale bar is 5 μm.

We used the GF-specific GAL4 enhancer-trap line, *A307 *[GAL4] (hereafter referred to as *A307*), to identify alterations of the GF morphological phenotype, brought about by Gl^DN ^over-expression, in the presence of the dominant enhancers or suppressors (Figure 3, Table 1). Our previous work indicated that there are two aspects to the phenotype observed when Gl^DN ^is targeted to the GF. First, the distinctive terminal bend does not form after 48 hrs of pupal development indicating that synaptogenesis with the TTMn is defective. Second, as the GF axon develops during later pupal stages, the tip swells, often to several times the axon diameter, presumably because of a build-up of cellular material that the motor is unable to move toward the cell body [20]. Like many GAL4-generated phenotypes there is some variation between preparations, most notably on the extent of axon swelling (compare Figure 3C and 6B). While both the lack of terminal bend and axon swelling are a result of retrograde motor disruption, any link between the two phenotypes is unclear. Both SG13/+ and SG46/+ showed suppression of these two phenotypes when Gl^DN ^was expressed using *A307 *(Figure 3F &3H, Table 1). Swollen axon tips were rarely seen and the axons were either "bendless," or showed at least one bend in a preparation (Figure 3F [arrowhead] &3H, Table 1), a phenotype not seen when the dominant-negative subunit is expressed alone (Table 1). SG58/+ showed a mild suppression of the GF morphology phenotype with consistently fewer neurons exhibiting swollen axons (Table 1, see below). However no bends or partial bends were observed (Figure 3G). We could not unequivocally rule out the possibility of these mutations altering the effectiveness of the GAL4/UAS system, however, the phenotypes observed for SG13/+ and SG46/+ were different from those seen if GAL4 expression was suppressed by reducing temperature (M.J.A unpublished observations). SG16/+ showed no rescue of the GF morphology phenotype, suggesting that this mutation does not interact with *Glued *in the GFS. The EG162/+ and EG165/+ caused dominant lethality in the *A307*>Gl^DN ^background (Table 1) presumably because they affected other cells expressing Gl^DN ^in the A307 line that eliminated viability. 20% (4/20) of EG28/+ preparations exhibited GF axons which remained in the brain (Figure 3E). This is likely to be due to a failure to exit the brain on outgrowth, or retraction after a failure in synatogenesis. No obvious enhancement of the GF phenotype was observed with EG37/+ (Figure 3D).

**Table 1:** Effects of enhancers and suppressors on GFS morphology

		GF morphology in *A307*>Gl^**DN **^background		
**Enhancer or suppressor**	***n*†**	**Swollen "bendless" axons (%)**	**"Bendless" axons (%)**	**Partial or "Wild type" bends (%)**

None	*29*	86	14	0
EG28/+	*38*	97*	3	0
EG37/+	*24*	92	8	0
EG162/+	*-*	Lethal	Lethal	Lethal
EG165/+	*-*	Lethal	Lethal	Lethal
SG13/+	*42*	0	17	83
SG16/+	*32*	88	12	0
SG46/+	*40*	23	32	45
SG58/+	*43*	65	35	0

		**GF morphology in *Gl*^**1**^/+ background**		

**3rd C'some enhancer**	***n†***	**Swollen "bendless" axons (%)**	**"Bendless" axons (%)**	**Partial or "Wild type" bends (%)**

None	*36*	0	8	92
EG7/+	*24*	0	25	75
EG28/+	*32*	0	3	97
EG56/+	*23*	0	13	87
EG79/+	*22*	0	18	82

†GFs were scored individually since the two neurons within one CNS could have different phenotypes.

* some axons failed to exit the brain (see figure 3F).

We reasoned that mutations in genes involved in synaptogenesis may not greatly enhance the already severe Gl^DN ^synaptic phenotype. Mutations in genes that have a role earlier in development, for example in axon guidance, may give rise to a discernable enhancement, such as incorrect GF axon growth as was the case for EG28/+. In *Gl*^1 ^mutants the GFs look morphologically normal, although they have GF-TTMn synaptic defects [20]. Therefore, we tested for dominant effects of the EG mutations on the morphology of the GFs in a *Gl*^1^/+ background by generating flies with *A307*, UAS-LacZ, *Gl*^1 ^and our third chromosome EGs (Figure 3I-K, Table 1). We saw an increase in the frequency of "bendless" axons in 3 of 4 EG mutations tested, suggesting an enhancement of the *Gl*^1^/+ phenotype, but no appearance of the swellings seen with over-expression of the dominant-negative subunit. Unusually, preparations containing EG28/+ and *Gl*^1^/+ exhibited a reduction in "bendless" axons and often had ectopic axonal branching (Figure 3K). Taken together with the phenotype seen with Gl^DN ^(see above), we presume this mutation is at a locus that has a role in axon growth and/or guidance. We were unable to do this with the mutations on chromosome two, using any GAL4 GF marker lines, since the mutations are on the same chromosome as the UAS-Gl^DN ^insert resulting in over-expression of Gl^DN ^in any cell that express GAL4 and contain the mutation. The UAS-Gl^DN ^element and the interacting mutations would need to be separated onto different chromosomes by either recombination or precise excision of the P-element containing the UAS-Gl^DN ^transgene. As an alternative, morphology of the GF can be observed using dye-filling techniques [31-33]. However, these techniques are labor-intensive and were not practicable for screening purposes.

### Mutations that interact with *Glued *in the Giant Fiber System and alter synaptic function

**Figure 4:**
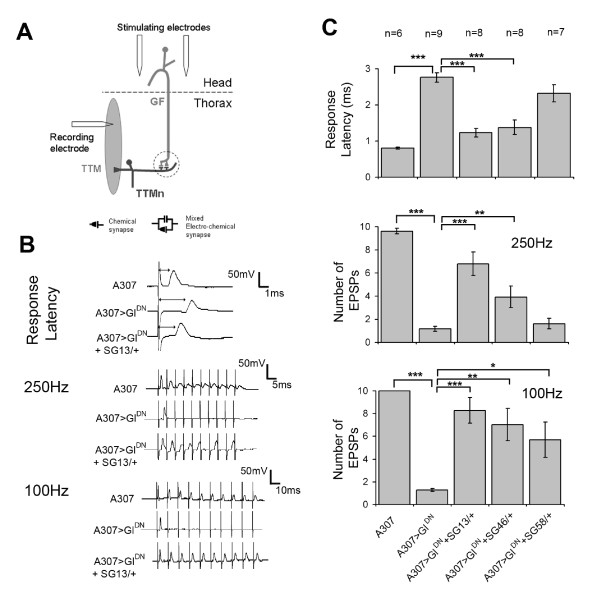
**Mutations that suppress the electrophysiological phenotype seen in *A307*>Gl^DN ^adult flies**. (A) Schematic depicting the GF, TTMn and the positioning of the stimulating and recording electrodes to test the function of the GF-TTMn synapse (circled). (B) Traces from individual flies showing the response latency upon a single stimulus and following to 10 stimuli at 250 and 100 Hz. A fly containing *A307 *alone shows a short response latency and 1:1 following at 250 and 100 Hz. A fly expressing Gl^DN ^exhibits a longer latency and gives only a single response or follows poorly at either 100 or 250 Hz. The addition of SG13/+ reduces the response latency back toward that of the control fly and increases following to stimuli at both 250 and 100 Hz. (C) Histograms showing the average response latencies and following to 10 stimuli at the two frequencies for the three suppressors tested. As indicated, responses from *A307*>Gl^DN ^flies were compared to *A307*-containing flies and all others were compared to *A307*>Gl^DN ^flies. *P < 0.05, **P < 0.01, ***P < 0.001 in unpaired Student's t-test.

We used electrophysiology to assay GFS function, which, in combination with morphological analysis, can reveal abnormalities of synaptogenesis during development [4-7,9,11,12,20]. A severe GF-TTMn synaptic phenotype is caused by over-expression of Gl^DN ^; some flies fail to respond to brain stimulation and those that do respond exhibit a long response latency and show only a single response or poor following to repetitive stimuli ([20]; Figure 4). We put the SG mutations which had exhibited morphological rescue into the *A307*/UAS-Gl^DN ^background. Corresponding with the observed morphological suppression (Figure 3F &3H, Table 1), flies also containing either SG13/+ or SG46/+ exhibited an increase in GF-TTMn synaptic function. All preparations responded to brain stimulation and had shorter response latencies and increased following at 100 and 250 Hz than is seen with simple over-expression of Gl^DN ^(Figure 4). SG58/+ also exhibited a detectable increase in synaptic function with all preparations responding upon stimulation, a slight reduction in the long latency seen when Gl^DN ^is over-expressed and also a corresponding increase in following to repetitive stimuli which was significant at 100 Hz (Figure 4).

**Figure 5:**
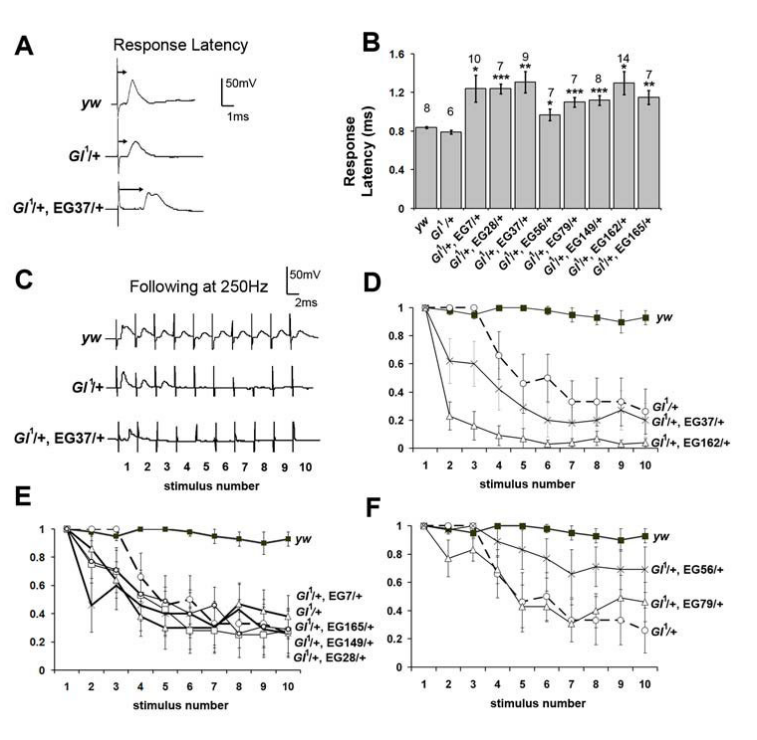
**Mutations that enhance the electrophysiological phenotype seen in *Gl*^1^/+ adult flies**. (A) Traces from individual flies showing the response latency upon a single stimulus. Both *yw *controls and *Gl*^1^/+ flies show short response latencies. The addition of EG37/+ into the *Gl*^1 ^background causes an increase in the latency. (B) Histograms showing the average response latencies recorded for all enhancers in the *Gl*^1 ^background. The number of preparations tested (*n*) is given above each bar and latencies for flies containing *Gl*^1^/+ plus enhancers compared to flies containing *Gl*^1^/+ alone. *P < 0.05, **P < 0.01, ***P < 0.001 in unpaired Student's t-test. (C) Traces from individual flies showing following to 10 stimuli at 250 Hz. *yw *controls usually gave a response to each stimulus (1-10) whereas this was depressed in *Gl*^1^/+ individuals which often gave a response to stimuli 1-3 and then failed to respond to the remaining 7 stimuli. The addition of EG37/+ enhances this effect. (D-F) Graphs showing the average probability of a response for stimuli 1 through 10 at 250 Hz. The data for *yw *and *Gl*^1^/+ flies is shown in each panel for comparison against flies trans-heterozygous for *Gl*^1^/+ and the various enhancers. Enhancers EG37/+ and EG162/+ seemed to increase the depression seen in *Gl*^1^/+ flies with a decrease in the probability of obtaining a response with subsequent stimuli (D). Other enhancers either had no effect (E) or slightly increased the probability of a response from the late (7-10) stimuli (F).

*Gl*^1^/+ mutants show a functional defect in the GF-TTMn synapse when tested using electrophysiology. They exhibit a response latency not significantly different from wild type (χ = 1.1 ms, *n *= 8) to brain stimulation, but do not follow 1:1 on stimulation at a frequency of 250 Hz as observed in wild type flies ([20]; Figure 5A &5C). We crossed the EGs into the *Gl*^1^/+ background to observe any enhancement of this synaptic phenotype. Because this test did not rely on GAL4 expression we were able to assay any of the EG mutations, regardless of their chromosomal location. All of the EGs tested showed significantly increased reponse latencies (Figure 5B) and exhibited poor following. To analyse this more carefully we looked at the probability of a response being seen with each sequential stimulus at 250 Hz. When 10 stimuli were given to *Gl*^1^/+ mutants, the flies demonstrated a depression in response to stimuli 1 through 10 with a plateau after stimulus 6 (Figure 5D), probably due to TTMn not always reaching threshold. This resulted in a 30% (probability 0.3) chance of responding to the last 4 stimuli for *Gl*^1^/+ flies whereas wild type flies will typically have > 90% (probability > 0.9) chance of responding (Figure 5D). When EG37/+ or EG162/+ were trans-heterozygous with *Gl*^1^/+ an enhancement of the phenotype was seen since 100% of *Gl*^1^/+ flies responded to stimulus number 3 whereas the probability of responding fell to 0.6 with EG37/+ and <0.2 with EG162/+ added (Figure 5D). We used root mean square deviation (RMSD) to measure the differences in the depression curves and compared them to that seen with *Gl*^1^/+. The average deviation between *Gl*^1^/+; EG37/+ and *Gl*^1^/+ was 0.23 (P < 0.1) and between *Gl*^1^/+; EG162/+ and *Gl*^1^/+ was 0.48 (P < 0.001). For most of the other enhancers tested there was no significant enhancement of the depression seen with *Gl*^1^/+ alone (Figure 5E). This included EG28/+ which had given outgrowth/retraction phenotypes when combined with Gl^DN ^(see Figure 3). Two of the EGs, EG56/+ & EG79/+, seemed to suppress the depression (Figure 5F) despite the fact that they caused an increase in the reponse latency (Figure 5B). The reason for this is not known.

### *aPKC*, but not *Su(H)*, interacts with *Glued *in synapse formation

**Figure 6:**
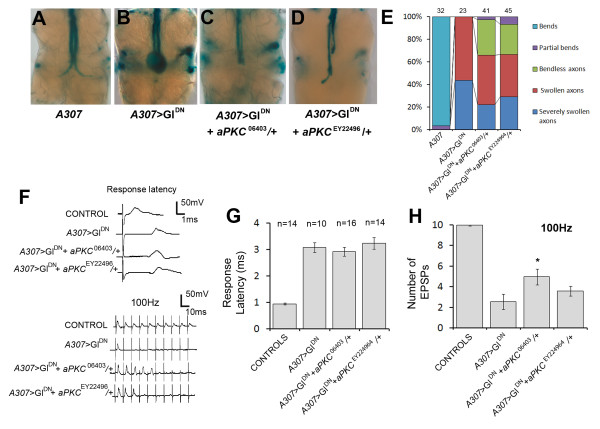
**Mutations in *aPKC *suppress the *A307*>Gl^DN ^phenotype**. (A-D) Dissected adult nervous systems stained for LacZ expression. Addition of a copy of either *aPKC *allele to the *A307*>Gl^DN ^background (C&D) suppresses the swollen and bendless axon tips seen in *A307*>Gl^DN ^preparations (B). (E) Quantification of the morphological phenotypes revealing the extent of suppression. Numbers of GFs scored are given above bars. Severely swollen axons were those that were > 3 times the normal diameter. (F) Traces from individual flies showing the response latency upon a single stimulus and following to 10 stimuli at 100 Hz in controls, A307>Gl^DN ^flies, and those also carrying the *aPKC *alleles tested. (G&H) Histograms showing the average response latencies and following at 100 Hz for the same genotypes. *P < 0.05 in unpaired Student's t-test. Controls were a mixture of UAS-Gl^DN^, *aPKC*^06403^/+ and UAS-Gl^DN^, *aPKC*^EY224964^/+ flies that did not carry *A307*.

We mapped the six mutations that showed significant interactions with *Glued *in the GFS using deficiencies and known lethal alleles (see materials and methods). This was assuming that the lethality was associated with the enhancer or supressor. A caveat being that lethality could be due to a second mutation and the interaction with *Glued *due to a viable allele. Two of the mutations mapped to known genes, *aPKC *and *Su(H)*, and the others were mapped to small regions on chromosome two or three (Table 2). To determine whether the suppression of the Gl^DN ^phenotype in the GF by SG58 was due to the mutation in *aPKC *we recombined both the *aPKC*^06403 ^and *aPKC*^EY22496 ^alleles onto the UAS-Gl^DN ^chromosome and crossed these lines to *A307*. Morphological examination of the GF axons revealed that they were less swollen than seen in *A307*>UAS-Gl^DN ^preparations and occasionally were seen to have a bend (Figure 6). GF-TTMn synaptic function was also increased compared to *A307*>Gl^DN ^flies. When tested using electrophysiology, 89% (16/18) of *A307*>Gl^DN^, *aPKC*^06403^/+ flies responded upon GF stimulation compared to 66% (10/15) of *A307*>Gl^DN ^flies and they exhibited a slightly reduced latency (Figure 6F &6G) and a statistically significant increase in following at 100 Hz (Figure 6F &6H). With the *aPKC*^EY22496 ^allele, weaker suppression was seen with 78% (14/18) responding and they exhibited an increase in following at 100 Hz (Figure 6F &6H). Overall, the results show weak support for the *aPKC *interaction with *A307*>UAS-Gl^DN ^from the *aPKC*^06403 ^allele and weaker support from the *aPKC*^EY22496 ^allele, however, they are very similar to the effect of the SG58/+ (Figures 3 &4) and indicate that *aPKC *interacts with *Glued *genetically to alleviate the phenotype caused by the over-expression of the poison subunit.

**Table 2:** Location of mutations isolated in the screen

Mutation	Chromosomal location or gene
SG13	60F5
SG46	22F
SG58	*aPKC*
EG28	84F7-12
EG37	*Su(H)*
EG162	46D-E

To determine whether the enhancement of the *Gl*^1^/+ phenotype in the GF by EG37/+ was due to the mutation in *Su(H) *we generated *Su(H)*^1^/+; *Gl*^1^/+ double heterozygotes and again looked at both the morphology of the GFs, with our *A307 *line, and performed electrophysiology to assay the function of the GF-TTMn synapse. No enhancement of the *Gl*^1^/+ phenotype was observed in these flies (data not shown). We cannot therefore rule out the presence of a second unmapped mutation on the EG37 chromosome. Alternatively, the mutation in EG37 may have different properties than the *Su(H)*^1 ^allele.

## Discussion

The success of our two-stage screening approach may have been facilitated by the fact that *Glued *has a plethora of distinct roles during eye development, including organizing optic neural architecture [17,34,35] and an involvement in the formation of sensory neuronal circuits [18,19]. Therefore we had an eye phenotype on which to base the screen. However, this does not preclude such a method being used for identifying genes involved in other aspects of neural differentiation. We found that 50% (6/12) of the isolated mutation-containing chromosomes that altered the eye phenotype also altered GFS phenotypes when tested.

The over-expression of the truncated Glued protein caused strong phenotypes in both the eye and GF neurons, greater than those caused by heterozygosity for the dominant *Gl*^1 ^allele. This is likely to be due to the GAL4-UAS system producing many more molecules of the truncated product than *Gl*^1^/+ cells in which, theoretically, a maximum of half of the Glued molecules will be truncated. Consistent with this observation, both the suppressors and enhancers isolated during this screen showed stronger effects on Gl^DN ^eye phenotypes than on those produced by *Gl*^1^. Determining interactions with the *Gl*^1^allele also allowed us to confirm GAL4-independent interactions with the *Glued *locus. For all of the mutations (with the exception of EG162), the alterations of the weaker *Gl*^1^/+ eye phenotype were not obvious, however, SEM and sectioning was performed to show interactions with two of the mutations (EG37 & SG13).

We used two different disruptions of Glued function, one strong and the other weaker, to assay successfully the effects of both enhancer and suppressor mutations in the GFS using both morphological and electrophysiological criteria. The severe disruptions of GF morphology and synaptic function enabled the effects of suppressor mutations to be clearly observed. This was less reliable when assaying the effects of mutations isolated as enhancers as either no increase of the already severe phenotype was seen or the interaction was lethal. For the enhancers, therefore, we relied on generating double heterozygotes with *Gl*^1^/+. As was the case in the eye, interactions were less pronounced and only two enhancers, EG37/+ and EG162/+ showed enhancement of the *Gl*^1^/+ electrophysiological phenotype. Indeed, the subtlety of some interactions with *Gl*^1^/+ may have resulted in our analyses being unable to detect some positive interacting loci in the GFS that altered the eye phenotype caused by Gl^DN^.

We have generated some EMS alleles, two of which we have mapped to known genetic loci (see below) and four of which we have mapped to discrete chromosomal locations (Table 2). However, these four complement all the available lethal alleles in these regions indicating that our mutations lie in loci for which there are few or no lethal alleles available. Identification of the location of these new alleles will require either new rounds of mutagenesis, such as via P-element excision in the mapped regions, finer mapping using SNPs [36-38] or custom made deficiencies using stocks from the DrosDel project [39]. Completion of the BDGP Gene Disruption Project may also enable mapping of the lesions [40] along with more recent approaches using other transposable elements that may disrupt genes refractory to P-element disruption [41-43]. Interestingly, we appear not to have isolated any mutations in genes that encode known components of the retrograde motor complex including any further alleles of *Glued*. During some of the early genetic analysis of the *Glued *locus, dominant second-site suppressors of the *Gl*^1 ^eye phenotype were isolated and reported [27]. Of these, two were mapped to the X chromosome (*Su [Gl]27 *&*Su [Gl]57*,[27]) and the others, *Su(Gl)77 *&*Su(Gl)102 *are alleles of *Dynein heavy chain 64C *[44,45]. From the map positions of our mutations, we have not re-isolated similar alleles. Because our primary screen involved making only the eye mutant for *Glued*, we could potentially isolate mutations that are lethal in combination with *Gl*^1 ^and would, therefore, not have been isolated Harte and Kankel's screen. However, none of the enhancers tested were lethal with *Gl*^1^.

We have successfully isolated two new alleles of known genes, *Su(H) *and *aPKC*. Of the two, we have shown that alleles of *aPKC *genetically interact with *Glued *in the GFS and suppress the abnormalities in GF-TTMn synapse formation seen when the retrograde motor complex is compromised by Gl^DN^. These abnormalities are: a lack of the presynaptic "bends"; a branching event that takes place after the two neurons have met [31]; swollen axon tips and a weak or absent functional synapse [20]. aPKC is part of a protein complex, with PAR-3 (Bazooka in *Drosophila*) and PAR6 that regulates cell polarity in a number of different tissues/cells of *Drosophila *and vertebrates including neurons [46-48]. So what is the role of *aPKC *in the GF neuron? In vertebrate neurons aPKC is needed for neurite outgrowth [49-51]. In contrast, aPKC in flies is an essential part of the machinery that polarizes dividing neuroblasts [52] but is not needed postmitotically for outgrowth [53]. Our data also indicate that aPKC is not needed for neurite extension since the introduction of *aPKC *mutations into our sensitized background has no effect on GF outgrowth. *aPKC *is involved in memory formation in *Drosophila *[54] and at the developing larval NMJ it regulates microtubules (MTs) both pre- and postsynaptically during synapse formation [55]. Indeed MTs are one of the major targets of the PAR-3/PAR-6/aPKC complex in several contexts [56-58]. aPKC regulates MT orientation in fibroblasts [59,60] and MT organization in the early embryo [61]. At the NMJ it controls MT stability with a reduction in aPKC activity causing a decreased association of MTs with the microtubule associated protein Futsch and MT fragmentation [55]. Dynein-dynactin is known to be involved in MT organization during growth cone remodeling as well as polarizing MTs in axons [62,63]. Our data indicate that dynein-dynactin and aPKC are acting antagonistically during formation of the GF presynaptic structure and suggest that both are needed to control microtubule organization and dynamics in synapse formation but have opposing roles. One simple explanation is that one of the roles of dynein-dynactin in the GF is to alter MT dynamics at the tip of the axon, when it has reached its post-synaptic target, so that they are more mobile enabling the presynaptic bend to be formed. aPKC regulates the stability of MTs thereby confining axon branching to a single bend. Blocking dynein-dynactin function prevents the MT re-organization needed for formation of the bends and this is ameliorated when aPKC function is reduced.

## Conclusion

We have used a novel approach to screen for genes involved in central synapse formation by performing a primary screen, using a sensitized background, on the adult eye and then a secondary screen, on the isolated mutations, for synaptic phenotypes. This study shows that forward genetic screens are powerful tools for identifying genes with roles in CNS development.

## Methods

### Drosophila strains

The *A307 *[GAL4] enhancer-trap line and the line containing the UAS-Gl^DN ^transgene on chromosome 2 (UAS-Gl^Δ96B^) have been described previously [20,33,64], as has the eye specific GMR-GAL4 line [65]. Stocks containing balancers, deficiency stocks, lethal alleles, *aPKC*^06403^, *aPKC*^EY22496 ^and *Su(H)*^1 ^flies were obtained from the Bloomington Drosophila Stock Center, Indiana, USA.

### Gl^DN ^over-expression screen

*w*; UAS-Gl^DN ^males, isogenized on the second and third chromosomes, were mutagenized by feeding overnight with a 0.25 mM solution of EMS in 1% sucrose. This dose was to generate, on average, only one lethal mutation per genome to facilitate downstream analysis. The mutagenized males were then mated to *w*; GMR-GAL4; *TM6B*/*MKRS *virgin females and the eyes of the progeny were scored for an enhancement or suppression of the Gl^DN ^eye phenotype. We isolated 324 adults with altered eye morphology; 215 potential enhancers and 109 potential suppressors. Mutations were recovered by mating flies with altered eye phenotypes individually to *w*; *CyO*/Sco; *MKRS*/*TM6B *flies. Sibling crosses were then performed to obtain lines with recessive lethal, or recessive viable, mutations on chromosomes 2 or 3 balanced over *CyO*, *MKRS *or *TM6B*. One hundred and sixteen lines were recovered with either a recessive lethal on chromosome 2, 3, or both. We did not obtain any recessive mutations that gave a homozygous visible phenotype. The 116 lines were crossed back to *w*; GMR-GAL4; *TM6B*/*MKRS *flies to confirm that the recovered mutations altered the GMR-GAL4/UAS-Gl^DN ^eye phenotype. This resulted in 26 lines containing mutations that enhanced the phenotype and 15 lines that suppressed the phenotype.

### Scanning electron microscopy (SEM) and Retinal sectioning

Whole flies were fixed in 2% glutaraldehyde and dehydrated in acetone. Samples were dried in a Polatron E3000 critical point dryer, mounted onto stubs, and coated with gold. Micrographs were taken on a Hitachi S-430 electron microscope. For retinal sections, head cases were dissected from whole flies and fixed in 2% glutaraldehyde, 2% paraformaldehyde in 0.1 M PBS (0.1 M NaCl, 0.1 M Na_2_HPO_4_/NaH_2_PO_4 _[pH 6.8]) overnight at 4°C. Following dehydration in an acetone series, the head cases were embedded in Durcupan resin and 2.5 μm sections cut with a Lieca: Jung RM2065 microtome. Sections were stained with Toluidine blue and photographed on a Leica DMR microscope using a Leica DC500 digital camera.

### CNS Histochemistry

The central nervous systems were dissected from adult flies in 0.1 M PBS with 0.05% Triton X-100. For X-Gal staining they were then fixed in 1% gluteraldehyde for 5 min. Preparations were washed in PBT (0.1 M PBS, 0.1% Triton X-100) and pre-warmed in 2 mls of X-Gal staining solution (3 mM K_4 _[Fe(CN)_6_], 3 mM K_3 _[Fe(CN)_6_], 1 mM MgCl_2_, 150 mM NaCl, 10 mM Na_2_HPO_4_/NaH_2_PO_4 _[pH 7.2], 0.3% Triton X-100) in a watch glass for 5 min at 37°C. To this was added 1 ml of staining solution, saturated with dissolved X-Gal (5-bromo-4-chloro-3-indolyl β-D-galactopyranoside) and pre-warmed to 37°C, and the preparations were incubated for several hours until staining of the GFs was complete.

### Electrophysiology

Recordings from the GFS of adult flies were made essentially as described in [20]; a method based on those described by [66] and [67]. Flies were cooled on ice until they were immobile and secured in wax, ventral side down, with the wings held outwards in the wax. The GFs were stimulated using a Grass S48 stimulator to deliver a 40 V pulse for 0.03 ms through tungsten electrodes pushed through the eyes and into the brain. A tungsten earth wire was placed into the abdomen. Glass microelectrodes (resistance 40-60 MΩ), filled with 3 M KCl, were driven through the cuticle into the TTM and DLM muscles to record responses. These were amplified using Getting 5A amplifiers (Getting Instruments, USA) and the data digitized using an analogue-digital Digidata 1320 and Axoscope 9.0 software (Axon Instruments, USA). A single pulse was delivered for response latency measurements and trains of 10 stimuli, either 4 ms (250 Hz) or 10 ms (100 Hz) apart, were given with a 5 s interval between each train for following frequency recordings. We routinely recorded from both TTM and a DLM in each preparation to ensure that correct stimulation of the GF was achieved. However, only data from the TTM are presented in this report.

### Genetic mapping

Mutations were mapped using the "classic"second chromosome "deficiency kit" (DK2) of 110 stocks from the Bloomington Drosophila Stock Center containing mostly deletions that were defined cytologically. Once deficiencies were identified that failed to complement the isolated lethal mutations, smaller deficiencies in the regions were tested for complementation including molecularly defined deficiencies recently made available from Exelixis and the DrosDel project [39,68]. Standard complementation tests were then performed with known lethal mutations in the chromosomal regions uncovered by the deficiencies. SG13 failed to complement *Df(2R)kr10 *but complemented *Df(2R)gsb *and *Df(2R)kr14 *indicating that SG13 lies at 60F5. SG46 failed to complement *Df(2L)79b *which covers 22A2 to 22E1. Testing smaller deletions within this region revealed that SG46 failed to complement the molecularly defined deletions *Df(2L)Exel17008 *(22B8;22D1) and *Df(2L)Exel17011 *(22E1;22F3). However when crossed together the two deletions failed to complement each other and both fail to complement alleles of *Dpp *which lies at 22F1-2. Therefore they both delete at least the region 22E1 to 22F3 and *Df(2L)Exel17008 *is incorrectly annotated. SG58 failed to complement *Df(2R)KnSA4 *and *Df(2R)XTE-58 *indicating it resided between 51D1 and 51D6 on chromosome 2. Using the available lethal mutations we determined SG58 to be an allele of *Drosophila *atypical Protein Kinase *C *(*aPKC*) as it failed to complement *aPKC*^06403 ^and *aPKC*^EY22496^, both known null alleles [52]. EG28 failed to complement *Df(3R)p13 *which extends from 84F1 to 85A8. Within this region EG28 failed to complement *Df(3R)exel6417 *but complemented *Df(3R)dsx29 *making its location to be within 84F7-84F12. All available lethal mutations were found to be not allelic to EG28. EG37 failed to complement *Df(2L)TE35BC-24 *and *Df(2L)A48 *and testing known lethal alleles uncovered by both these deficiencies revealed the mutation to be in the *Su(H) *locus. EG37 is lethal when trans-heterozygous with the *Su(H)*^1 ^allele. EG162 failed to complement *Df(2R)X1 *and *Df(2R)stan1 *indicating that is resides between 46D and 47A. However, it did complement *Df(2R)stan2 *indicating that it lies at 46D-E. We have been unable to map this mutation further at present.

## Authors' contributions

LM and LAJ carried out the genetic screen; LAJ performed the SEM and eye sectioning; LM and MJA did the electrophysiology, all authors performed CNS dissection and staining. MJA designed and coordinated the work and drafted the manuscript. All authors read and approved the final manuscript.
